# Leishmanicidal Activity and Structure-Activity Relationships of Essential Oil Constituents

**DOI:** 10.3390/molecules22050815

**Published:** 2017-05-16

**Authors:** Audrey R. S. T. Silva, Ricardo Scher, Flaviane V. Santos, Sebastião R. Ferreira, Sócrates C. H. Cavalcanti, Cristiane B. Correa, Lilian L. Bueno, Ricardo J. Alves, Damião P. Souza, Ricardo T. Fujiwara, Silvio S. Dolabella

**Affiliations:** 1 Department of Pharmacy, Federal University of Sergipe, 49100-000 São Cristóvão, Sergipe, Brazil; audreytavares2@gmail.com (A.R.S.T.S.); socratescavalcanti@yahoo.com.br (S.C.H.C.); 2 Laboratory of Molecular Biology of Pathogenic Bioagents, Department of Morphology, Federal University of Sergipe, 49100-000 São Cristóvão, Sergipe, Brazil; scher@ufs.br (R.S.); flavianevieirah@gmail.com (F.V.S.); crisbani@gmail.com (C.B.C.); 3 Department of Parasitology, Biological Sciences Institute, Universidade Federal de Minas Gerais, 31270-901 Belo Horizonte, Minas Gerais, Brazil; ferreirasr2008@hotmail.com (S.R.F.); lilacerdabueno@gmail.com (L.L.B.); fujiwara@icb.ufmg.br (R.T.F.); 4 Health Science Center, Federal University of Roraima, Av. Cap. Ene Garcez, 69310-000 Boa Vista, Roraima, Brazil; 5 Department of Pharmaceutical Products, Federal University of Minas Gerais, 31270-901 Belo Horizonte, Minas Gerais, Brazil; ricardodylan@farmacia.ufmg.br; 6 Department of Pharmaceutical Sciences, Federal University of Paraíba, 58051-900 João Pessoa, Paraíba, Brazil; damiao_desousa@yahoo.com.br; 7 Laboratory of Parasitology and Tropical Entomology, Department of Morphology, Federal University of Sergipe, 49100-000 São Cristóvão, Sergipe, Brazil

**Keywords:** monoterpenes, *Leishmania amazonensis*, leishmanicidal activity, essential oil

## Abstract

Several constituents of essential oils have been shown to be active against pathogens such as bacteria, fungi, and protozoa. This study demonstrated the in vitro action of ten compounds present in essential oils against *Leishmania amazonensis* promastigotes. With the exception of *p*-cymene, all evaluated compounds presented leishmanicidal activity, exhibiting IC_50_ between 25.4 and 568.1 μg mL^−1^. Compounds with the best leishmanicidal activity presented a phenolic moiety (IC_50_ between 25.4 and 82.9 μg mL^−1^). Alicyclic alcohols ((−)-menthol and isoborneol) and ketones ((−)-carvone) promoted similar activity against the parasite (IC_50_ between 190.2 and 198.9 μg mL^−1^). Most of the compounds showed low cytotoxicity in L929 fibroblasts. Analysis of the structure-activity relationship of these compounds showed the importance of the phenolic structure for the biological action against the promastigote forms of the parasite.

## 1. Introduction

Leishmaniasis is a disease of worldwide distribution, present in Africa, Latin America, Asia, and Europe. It is estimated that 1.3 million new cases occur annually in these continents, with approximately 20,000 to 30,000 deaths due to the disease [1]. Leishmaniasis is transmitted by sandflies and is usually associated with poverty, malnutrition, poor living conditions, and climate and environmental changes [1,2]. Leishmaniasis can be caused by different protozoa species of the genus Leishmania, and the development of the disease is influenced by factors such as parasite species, parasite-host interaction, and vulnerability of the host immune system, presenting variable clinical manifestations as cutaneous, diffuse cutaneous, mucocutaneous and visceral leishmaniasis [3,4].

Antimonials and amphotericin B are the first-choice drugs for the treatment of leishmaniasis in most countries. However, there are several limitations related to their use, such as high financial cost (drug price and hospital support/hospitalization), long term treatment (up to 30 days), high toxicity and variable efficacy (30 to 98% cure). In addition, the emergence of resistant strains, especially in endemic areas, is also a limiting factor for the use of traditional chemotherapies [5,6,7].

A vast array of essential oil-bearing plants has been used to control parasites for centuries [8,9]. For example, garlic oil is known to be active against 12 different human and nonhuman parasites [10], eugenol-containing basil and clove oils possess antiphagocytic activity, and *Mentha crispa* essential oil is active against *Trypanosoma brucei* [11]. The discovery of new drugs has been based on natural products for years, either by the synthesis of substances that mimic a natural product, by modifying an existing natural molecule, or by the use of the natural product itself [12]. Among natural products, monoterpenes stand out for their wide use in the industry, in addition to having vast biological activity already verified against fungi, bacteria, viruses, and parasites, including protozoa [13,14,15,16,17,18,19,20,21]. Thus, the present work aims to investigate the relationship between chemical structures and the activity of different monoterpenes and a phenylpropanoide, constituents of essential oils, against promastigote forms of *Leishmania amazonensis*.

## 2. Results and Discussion

Nine monoterpenes and one phenylpropanoid were selected (Table 1) to investigate the action of these compounds against *L. amazonensis* promastigotes and to determine the relationship between their chemical structure and biological activity.

Among the compounds evaluated in this study, three presented a phenolic moiety: carvacrol (IC_50_ 25.4 μg mL^−1^), thymol (IC_50_ 26.8 μg mL^−1^), and eugenol (IC_50_ 82.9 μg mL^−1^). The positional isomers carvacrol and thymol were the most active compounds in this series, exhibiting the lowest IC_50_ values among evaluated compounds. In addition, these compounds presented similar leishmanicidal activity, possibly suggesting that the different positions of the hydroxyl in the aromatic ring does not influence the antiparasitic activity.

The acidity of phenolic hydroxyl groups may play an important role in explaining higher potencies of phenolic compounds in this series. Aside from the hydroxyl and the aromatic ring, eugenol also bears a hydrogen bond between the methoxyl in the *ortho* position and the phenolic OH, which reduces the release of protons by the OH group due to the presence of this intramolecular hydrogen bonding [22]. This molecular property might justify eugenol lower potency in relation to both phenolic compounds deprived of intramolecular hydrogen bonds, carvacrol and thymol [23].

Non-aromatic hydroxylated compounds, such as isoborneol and menthol alcohols, exhibited weaker antiparasitic action than phenolic compounds (IC_50_ of 190.2 μg mL^−1^ and 198.0 μg mL^−1^, respectively). Within the aliphatic alcohols tested, linalool was the least active compound (IC_50_ of 276.2 μg mL^−1^). Carvone, an α,β-unsaturated ketone, exhibited similar results to isoborneol and menthol alicyclic alcohols (IC_50_ of 194.7 μg m^−1^). The enone of carvone might play an important antiparasitic activity, as expected from the hydroxyls of alcohols, once conjugated ketones have the potential to function as a Michael acceptor by reacting with nucleophilic species of the parasite [24].

The importance of the phenolic hydroxyl group as for leishmanicidal activity is observed in carvacrol, thymol, *p*-cymene, and menthol in a similar manner, as previously observed by Ultee et al. [25] for monoterpenes with antibacterial action. Interestingly, *p*-cymene, a precursor of thymol and carvacrol, which does not have the hydroxyl group, did not exhibit any activity against *L. amazonensis*, unlike the two hydroxylated derivatives that exhibited better results. These results suggest that the lack of a polar hydroxyl group in the *p*-menthane ring template, as well as the absence of aromaticity might render monoterpenes inactive. The importance of aromatic hydroxyls for antiparasitic activity is again highlighted in the analysis of menthol. Although menthol has a hydroxyl group, the absence of the aromatic ring contributed to an IC_50_ well above phenolic compounds. The importance of the aromatic structure for leishmanicidal activity was also observed in the study of synthetically modified lactones [26].

Previous studies of carvacrol in the literature found an IC_50_ of 2.3 μg mL^−1^ [27] and 28.0 μg mL^−1^ [28] against *L. chagasi* promastigotes, while another study found an IC_50_ of 15.3 μg mL^−1^ against *L. amazonensis* promastigotes [29]. The different values of IC_50_, when comparing the same species and compound, are probably related to the experimental design. In a previous study, the use of longer drug-parasite incubation (72 h) and the assay development performed with the chromogen *p*-nitrophenol phosphate to measure viability, determined the IC_50_ of 15.3 μg mL^−1^ for carvacrol [29]. Meanwhile, in the present study (IC_50_ of 25.4 μg mL^−1^), the parasites were incubated for 24 h using the resazurin method.

Several studies have reported the action of thymol on species of *Leishmania*, such as *L. chagasi* (IC_50_ of 9.8 μg mL^−1^, 12.85 μg mL^−1^, and 65.2 μg mL^−1^) [28,30,31], *L. panamensis* (IC_50_ of 194.3 µg mL^−1^) [32] and *L. amazonensis* (IC_50_ of 22.6 μg mL^−1^) [33], presenting similar activity to the ones observed in our study. Eugenol, on the other hand, was evaluated against promastigote forms of *L. chagasi* (IC_50_ of 56.1 μg mL^−1^) [30] and *L. amazonensis* (IC_50_ of 500 μg mL^−1^ and 80.0 μg mL^−1^) [34,35], and the latter exhibits similar results to our experiment.

The potent behavior of thymol and carvacrol identified in the present study, when compared with other monoterpenes, has already been observed previously in bacteria. Dorman and Deans [36] evaluated the action of six essential oils and their major compounds, with 21 monoterpenes and the phenylpropanoid eugenol, against 25 different bacterial species, and identified that thymol, carvacrol, and eugenol were much more potent than the other monoterpenes. These authors also discussed a value of the phenolic hydroxyl present in these compounds for this biological activity. Nazzaro et al. [37] still emphasized the importance of the delocalization of electrons, besides the phenolic hydroxyl, as important characteristics for the antibacterial activity of carvacrol and thymol. However, bacteria and protozoa are microorganisms, thus they are relatively different in structural and molecular terms.

Isoborneol, a bicyclic alcohol, was previously evaluated against promastigotes of *L. infantum*, *L. tropica*, and *L. major*. No leishmanicidal activity was found, even at a maximum dose of 400.0 μg mL^−1^ [38]. To date, there are no reports of isoborneol activity against *L. amazonensis*. In our study, isoborneol exhibited an IC_50_ of 190.2 μg mL^−1^ as leishmanicidal against *L. amazonensis*. In this study, linalool, an acyclic tertiary alcohol, exhibited an IC_50_ of 276.2 μg mL^−1^, considerably higher than the value of 4.3 ng mL^−1^ found by ROSA et al. [39], which was also obtained on *L. amazonensis* promastigotes. Meanwhile, Dutra et al. [35] did not identify changes in the viability of promastigotes of *L. infantum chagasi* treated with linalool up to the dose of 750 μg mL^−1^. These authors believe that the difference between their results and those observed by ROSA et al. [39] may be related to different concentrations of enantiomers. The literature does not mention the action of menthol, a cyclic alcohol, in *L. amazonensis* or other species of *Leishmania*. In our results, an IC_50_ of 198.0 μg mL^−1^ was obtained for this compound.

Among the monoterpenes, two hydrocarbons 3-carene and *p*-cymene were tested. No report was found in the literature of the leishmanicidal activity of the bicyclic hydrocarbon 3-carene on *L. amazonensis*. In this study, an IC_50_ of 72.5 μg mL^−1^ was found for 3-carene against *L. amazonensis*. However, on *L. donovani* promastigotes, 3-carene exhibited an IC_50_ of 27.0 μg mL^−1^ [40]. *p*-Cymene, an aromatic hydrocarbon, was evaluated on *L. chagasi* and found to have an IC_50_ of 149.1 μg mL^−1^ [28]. On the other hand, in our experiments, no effect on the viability of *L. amazonensis* promastigotes was found in concentrations up to 1000.0 μg mL^−1^.

(−)-Carvone was previously evaluated on *L. chagasi* promastigotes and exhibited an IC_50_ > 300.0 μg mL^−1^ [28]. However, no IC_50_ for carvone was found in the literature against *L. amazonensis*. In our trial, an IC_50_ of 194.7 μg mL^−1^ was obtained for (−)-carvone against *L. amazonensis*. Similar results were also obtained for the alicyclic alcohols isoborneol and menthol.

Machado et al. [38] evaluated the cyclic ether 1,8-cineole on promastigotes of *L. infantum*, *L. tropica*, and *L. major* in increasing doses up to 400 μg mL^−1^. However, no leishmanicidal activity was detected. On the other hand, Camargos et al. [41] obtained an IC_50_ of 4697.0 µM against *L. amazonensis* promastigotes, which is equivalent to 724.0 μg mL^−1^, similar to that obtained in the present study (568.1 μg mL^−1^).

The replacement of the hydroxyl group in menthol by the ether function found in the 1,8-cineole *p*-menthane ring resulted in a lower toxic effect (IC_50_ = 568.1 μg mL^−1^ as compared to 198.9 μg mL^−1^ of menthol), against the assessed species of *Leishmania*. This result corroborates the importance of the aromatic ring and the hydroxyl group to yield more potent compounds.

The interpretation of the results and comparison between studies should also consider similar species of *Leishmania* and life stage, such as promastigote or amastigote forms of the parasite, since different species of the parasite and evolutionary forms have specific biological characteristics that can produce discordant results [42,43,44]. In addition, the results are dependent on the experimental model being used [38].

In general, the compounds showed low cytotoxicity on L929 fibroblasts at the concentrations evaluated, 50.0 and 100.0 μg mL^−1^. The results of the tests are expressed as a percentage of viability (Table 2).

An intensity scale based on the methods of Rodrigues et al. [45] was used to classify the cytotoxicity of the compounds. At 50.0 μg mL^−1^, 3-carene, menthol and *p*-cymene were considered non-cytotoxic. The remaining compounds showed low cytotoxicity. At the concentration of 100.0 μg mL^−1^, only menthol was not considered cytotoxic. Most compounds showed low cytotoxicity, whereas carvacrol and 3-carene showed moderate cytotoxicity to the cells. Additionally, essential oil components, such as carvacrol, thymol, 1,8-cineole, isoborneol, menthol, carvone, linalool, and *p*-cymene are considered to be safe food additives, an indication of low mammalian toxicity [46].

The structure-activity relationships are important not only to understand how these compounds act on the parasite, but also to guide future studies on these molecules. Although most of the compounds used in this work had an IC_50_ higher than 100.0 μg mL^−1^, the tests are still preliminary, performed only on *L. amazonensis* promastigotes. Some studies show a greater sensitivity to intracellular amastigote forms of *Leishmania*, with an IC_50_ about 50% lower than that found for promastigotes [47,48]. Thus, a more detailed evaluation of these compounds in the intracellular form of the parasite and in other mammalian cells is needed to better evaluate the toxicity of these compounds.

## 3. Materials and Methods

### 3.1. Compounds

The following compounds were used: 3-carene (96.1% purity) (IUPAC (International Union of Pure and Applied Chemistry) name: 4,7,7-trimethylbicyclo[4.1.0]hept-3-ene), carvacrol (99.9%) (IUPAC name: 2-methyl-5-propan-2-ylphenol), (−)-carvone (99.4%) (IUPAC name: (5*R*)-2-methyl-5-prop-1-en-2-ylcyclohex-2-en-1-one), 1,8-cineole (99.7%) (IUPAC name: 2,2,4-trimethyl-3-oxabicyclo[2.2.2]octan-6-ol), *p*-cymene (99.7%) (IUPAC name: 1-methyl-4-propan-2-ylbenzene), isoborneol (99.0%) (IUPAC name (1*R*,3*R*,4*R*)-4,7,7-trimethylbicyclo[2.2.1]heptan-3-ol), (−)-linalool (99.1%) (IUPAC name: (3*R*)-3,7-dimethylocta-1,6-dien-3-ol) e (−)-menthol (99.4%) (IUPAC name: (1*R*,2*S*,5*R*)-5-methyl-2-propan-2-ylcyclohexan-1-ol) purchased from Sigma-Aldrich (St. Louis, MO, USA), eugenol (99.0%) (IUPAC name: 2-methoxy-4-prop-2-enylphenol) purchased from Biodinâmica (Ibiporã, PR, Brazil) and thymol (99.0%) (IUPAC name: 5-methyl-2-propan-2-ylphenol) purchased from Synth (Diadema, SP, Brazil), all with analytical purity.

### 3.2. Parasites

Culture of *L. amazonensis* (LTCP 9667 obtained by Giudice et al. [44]) promastigotes were maintained at 24 °C in Schneider′s Drosophila medium (Sigma-Aldrich, St. Louis, MO, USA; pH 6.7) supplemented with 10% (*v*/*v*) inactivated fetal bovine serum (FBS, Gibco by Thermo Fisher Scientific, Carlsbad, CA, USA), ampicillin 500 mg m^−1^, 1% and gentamicin 40 mg mL^−1^, 0.1% (Sigma-Aldrich, St. Louis, MO, USA).

### 3.3. Fibroblasts

Culture of the mouse fibroblast cell line (L929, ATCC CCL-1) were maintained in Dulbecco′s Modified Eagle Medium (DMEM, Sigma-Aldrich, St. Louis, MO, USA) supplemented with 10% heat-inactivated FBS and 1% streptomycin/penicillin (5000 units + 5 mg mL^−1^, Sigma-Aldrich, St. Louis, MO, USA), and kept in a humid atmosphere at 37 °C and 5% CO_2_.

### 3.4. Evaluation of Leishmanicidal Activity

Promastigotes of *L. amazonensis* in log-phase growth were distributed in a 96-well plate (5 × 10^5^ cells/well) and treated with the evaluated compounds solubilized in dimethyl sulfoxide (DMSO, Sigma-Aldrich, St. Louis, MO, USA) and diluted in Schneider′s medium in different concentrations (between 0.0 and 1000.0 μg mL^−1^) and incubated for 24 h at 24 °C in a biochemical oxygen demand (BOD) incubator. Promastigotes incubated in the absence of test compounds were used as a negative control. Promastigotes treated with Amphotericin B (Sigma-Aldrich, St. Louis, MO, USA) were used as a positive control. The cellular viability of the parasites was evaluated using the colorimetric method of resazurin, adapted from Kulshrestha et al. [49]. Briefly, after the treatment time, 50 μL of resazurin (2 mM mL^−1^ in phosphate-buffered saline (PBS)) (Sigma-Aldrich, St. Louis, MO, USA) was added per well and the plates were again incubated for 6 h at 24 °C, then read in a spectrophotometer (Synerg H1, Biotek, Winooski, VT, USA) at 570 and 595 nm. Absorbance was used to calculate cell viability based on the following equation:

(1) $$Viability\;\left(\%\right)=\frac{Abs.570\;nm-\left(Abs.595\;nm\;\times \;RO\right)\;test}{Abs.570\;nm\;–\left(Abs.595\;nm\;\;\times \;RO\right)\;control\;}\;\times \;100$$

wherein:

(2) $$RO=\;\frac{OD\;medium\;with\;resazurin\;in\;570\;nm-OD\;medium\;without\;resasurin\;in\;570\;nm}{OD\;medium\;with\;resazurin\;in\;595\;nm-OD\;medium\;without\;resazurin\;in\;595\;nm}$$

The IC_50_ values were obtained by a non-linear regression analysis from the viability values using the GraphPad Prism 5.0 program. All experiments were performed in triplicate from three independent experiments and the data were expressed as mean ± standard deviation (±SD).

### 3.5. Cytotoxicity

Fibroblasts were distributed in 96-well plates (2 × 10^4^ cells/well) and incubated for 24 h in a 5% CO_2_ atmosphere at 37 °C. After this period, the medium was removed and the adhered cells were treated with the compounds at concentrations of 50.0 and 100.0 μg mL^−1^ for 24 h under the same incubation conditions. Untreated cells were used as controls and considered with 100% cell viability. After the treatment period, cell viability was determined by MTT assay as described in ISO 10993-5 [50], with modifications. For this, the cell monolayer was washed twice with PBS (pH 7.4), and then 200 μl MTT (0.5 mg mL^−1^ in PBS, Sigma-Aldrich, St. Louis, MO, USA) was added to each well. The plates were again incubated under the same conditions as listed above, for a period of 3 h. After the incubation time, the MTT was aspirated and the formazan crystals were solubilized in 200 μL of DMSO. After 10 min, the optical density (OD) was measured on a microplate reader at the wavelength of 570 nm. The results were expressed as percentage of viability according to the following equation:

(3) $$Viability=\frac{Absorbance\;\left(treated\;cell\right)}{Absorbance\;\left(control\;cell\right)}\;\times \;100$$

Each experiment was conducted in quadruplicate and repeated at least three times. Data were expressed as mean ± standard deviation (±SD).

## 4. Conclusions

In this work, the compounds with phenolic moiety carvacrol, thymol, and eugenol were the most potent against *L. amazonensis* promastigotes. In addition, the evaluated compounds exhibited low cytotoxicity on L929 fibroblasts.

Studies with a clinically more relevant intracellular amastigote form will have to be conducted in order to further evaluate the potential of these compounds. Even though the overall activity level of the investigated compounds is low compared to amphotericin B, the results presented in this work demonstrate there are activity differences among the essential oil constituents distinguishing between more and less potent compounds that may serve as a basis for the planning of more promising antileishmanial agents.

## Figures and Tables

**Table 1:** In vitro leishmanicidal activity of different essential oils constituents against *Leishmania amazonensis* promastigotes.

Compound	Chemical Structure	IC_50_ (µg mL^−1^) *	IC_50_ (µM) *	R^2^
Carvacrol (1)	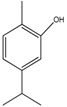	25.4 ± 2.4	169.08 ± 15.97	0.70
Thymol (2)	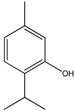	26.8 ± 3.7	178.40 ± 24.63	0.81
3-Carene (3)	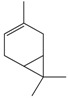	72.5 ± 18.5	532.18 ± 135.79	0.81
Eugenol (4)	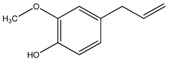	82.9 ± 6.2	504.87 ± 37.75	0.98
Isoborneol (5)	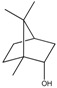	190.2 ± 9.8	1233.06 ± 63.53	0.97
(–)-Carvone (6)	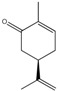	194.7 ± 16.9	1296.09 ± 112.50	0.94
(–)-Menthol (7)	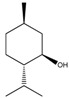	198.9 ± 12.0	1272.87 ± 76.79	0.96
(–)-Linalool (8)	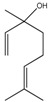	276.2 ± 24.0	1790.59 ±155.59	0.91
1,8-Cineole (9)	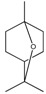	568.1 ± 56.5	3682.98 ± 366.28	0.89
*p*-Cymene (10)	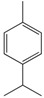	>1000	>7450.45	-
^c^ Amphotericin B	-	0.05 ± 0.01	0.054 ± 0.01	-

^a^ IC_50_ = Drug concentration capable of inhibiting 50% of promastigote multiplication; ^b^ R^2^ = Coefficient of determination—measure for the quality of the curve fitting of sigmoidal dose-response curves; ^c^ Amphotericin B was used as positive control. * Represents the mean of three independent experiments conducted in triplicate and expressed with means plus or minus standard deviation (±SD).

**Table 2:** Cytotoxic activity of the compounds on L929 fibroblasts.

Compounds	Viability (%)	
	50 µg mL^−1^	100 µg mL^−1^
3-Carene	84.1 ± 6.4 **	48.7 ± 6.7 *
Carvacrol	51.3 ± 3.0	46.1 ± 2.9 *
(−)-Carvone	65.1 ± 4.7	58.2 ± 4.2
1,8-Cineole	71.4 ± 0.7	66.9 ± 7.8
Eugenol	78.1 ± 7.0	63.1 ± 1.7
Isoborneol	73.3 ± 9.4	72.9 ± 7.5
(−)-Linalool	65.7 ± 8.2	66.7 ± 7.8
(−)-Menthol	83.8 ± 8.0 **	81.2 ± 2.4 **
Thymol	64.5 ± 4.0	58.5 ± 6.7
*p*-Cymene	91.2 ± 6.6 **	87.1 ± 6.7 **

Low cytotoxicity (viability between >50% and <80%); * Moderate cytotoxicity (viability between >30% and <50%); and ** Non-cytotoxic (viability >80%).

## References

[1] World Health Organization Leishmaniasis-Fact Sheet.

[2] Savoia D. (2015). Recent updates and perspectives on leishmaniasis. J. Infect. Dev. Ctries.

[3] Kevric I., Cappel M.A., Keeling J.H. (2015). New World and Old World *Leishmania*. Infections A Practical Review. Dermatol. Clin..

[4] Vries H.J.C., Reedijk S.H., Henk S.D.F.H. (2015). Cutaneous Leishmaniasis: Recent developments in diagnosis and management. Am. J. Clin. Dermatol..

[5] Ejazi S.A., Ali N. (2013). Developments in diagnosis and treatment of visceral leishmaniasis during the last decade and future prospects. Expert Rev. Anti Infect. Ther..

[6] Kobets T., Grekov I., Lipoldová M. (2012). Leishmaniasis: Prevention, parasite detection and treatment. Curr. Med. Chem..

[7] Goto H., Lindoso J.A.L. (2010). Current diagnosis and treatment of cutaneous and mucocutaneous leishmaniasis. Expert Rev. Anti Infect. Ther..

[8] Jones F.A. (1996). Herbs–useful plants. Their role in history and today. Eur. J. Gastroenterol. Hepatol..

[9] Bahmani M.P., Bahmani M., Shahsavari S., Naghdi N., Ezatpour B., Moradniani M., Rafieian-Kopaei M., Sari M. (2016). A review of the antiparasitic medicinal plants used in ethnobotany of different regions of Iran. Der. Pharm. Chem..

[10] Anthony J.P., Fyfe L., Smith H. (2005). Plant active components—A resource for antiparasitic agents?. Trends Parasitol..

[11] Sousa D.P., Cardoso T.L., Steverding D. (2016). Evaluation of antiparasitc activity of *Mentha. crispa* essential oil, its major constituent rotundifolone and analogues against *Trypanosoma brucei*. Planta Med..

[12] Newman D.J., Cragg G.M. (2016). Natural Products as Sources of New Drugs from 1981 to 2014. J. Nat. Prod..

[13] Mafud A.C., Silva M.P., Monteiro D.C., Oliveira M.F., Resende J.G., Coelho M.L., De Sousa D.P., Mendonça R.Z., Pinto P.L., Freitas R.M. (2016). Structural parameters, molecular properties, and biological evaluation of some terpenes targeting *Schistosoma. mansoni* parasite. Chem. Biol. Interact..

[14] Peixoto M.G., Costa-Júnior L.M., Blank A.F., Lima A.S., Menezes T.S.A., Santos D.A., Alves P.B., Cavalcanti S.C., Bacci L., Arrigoni-Blank M.F. (2015). Acaricidal activity of essential oils from *Lippia. alba* genotypes and its major components carvone, limonene, and citral against *Rhipicephalus. microplus*. Vet. Parasitol..

[15] Sánchez C., Aznar R., Sánchez G. (2015). The effect of carvacrol on enteric viruses. Int. J. Food Microbiol..

[16] Koziol A., Stryjewska A., Librowski T., Salat K., Gawel M., Moniczewski A., Lochyński S. (2014). An overview of the pharmacological properties and potential applications of natural monoterpenes. Mini Rev. Med. Chem..

[17] Reis S.L., Mantello A.G., Rossete E.A.G., Cardoso A.M., Beleboni R.O. (2014). Insecticidal and repellent activity of typical monoterpenes from plant essential oils against *Callosobruchus. maculatus* (Fabr. 1775). BMC Proc..

[18] Moraes J., Almeida A.A.C., Brito M.R.M., Marques T.H.C., Lima T.C., de Sousa D.P., Nakano E., Mendonça R.Z., Freitas R.M. (2013). Anthelmintic Activity of the Natural Compound (+)-Limonene Epoxide against *Schistosoma. mansoni*. Planta Med..

[19] Hui X., Yan G., Tian F.L., Li H., Gao W.Y. (2017). Antimicrobial mechanism of the major active essential oil compounds and their structure-activity relationship. Med. Chem. Res..

[20] Bakkali F., Averbeck S., Averbeck D., Idaomar M. (2008). Biological effects of essential oils–A review. Food Chem. Toxicol..

[21] Trombetta D., Castelli F., Sarpietro M.G., Venuti V., Cristani M., Daniele C., Saija A., Mazzanti G., Bisignano G. (2005). Mechanisms of Antibacterial Action of Three Monoterpenes. Antimicrob. Agents Chemother..

[22] Olbert-Majkut A., Wierzejewska M. (2008). Conformational Study of Eugenol by Density Functional Theory Method and Matrix-Isolation Infrared Spectroscopy. J. Phys. Chem. A.

[23] Arfa A.B., Combes S., Preziosi-Belloy L., Gontard N., Chalie P. (2006). Antimicrobial activity of carvacrol related to its chemical structure. Lett. Appl. Microbiol..

[24] Pizzolitto R.P., Herrera J.M., Zaio Y.P., Dambolena J.S., Zunino M.P., Gallucci M.N., Zygadlo J.A. (2015). Bioactivities of Ketones Terpenes: Antifungal Effect on *F. verticillioides* and Repellents to Control Insect Fungal Vector, *S. zeamais*. Microorganisms.

[25] Ultee A., Bennik M.H.J., Moezelaar R. (2002). The Phenolic Hydroxyl Group of Carvacrol Is Essential for Action against the Food-Borne Pathogen *Bacillus cereus*. Appl. Environ. Microbiol..

[26] Castaño M., Cardona W., Quiñones W., Robledo S., Echeverri F. (2009). Leishmanicidal Activity of Aliphatic and Aromatic Lactones: Correlation Structure-Activity. Molecules.

[27] Melo J.O., Bitencourt T.A., Fachin A.L., Cruz E.M.O., Jesus H.C.R., Alves P.B., Arrigoni-Blank M.F., Franca S.C., Beleboni R.O., Fernandes R.P.M. (2013). Antidermatophytic and antileishmanial activities of essential oils from *Lippia. gracilis Schauer.* genotypes. Acta Trop..

[28] Escobar P., Leal S.M., Herrera L.V., Martinez J.R., Stashenko E. (2010). Chemical composition and antiprotozoal activities of Colombian *Lippia.* spp essential oils and their major componentes. Mem. Inst. Oswaldo Cruz.

[29] Monzote L., Stamberg W., Staniek K., Gille L. (2014). Toxic effects of carvacrol, caryophyllene oxide, and ascaridole from essential oil of *Chenopodium. ambrosioides* on mitochondria. Toxicol. Appl. Pharm..

[30] Morais S.M., Vila-Nova N.S., Bevilaqua C.M.L., Rondon F.C., Lobo C.H., Moura A.A.A.N., Sales A.D., Rodrigues A.P., de Figuereido J.R., Campello C.C. (2014). Thymol and eugenol derivatives as potential antileishmanial agentes. Bioorg. Med. Chem..

[31] Farias-Junior P.A., Rios M.C., Moura T.A., Almeida R.P., Alves P.B., Blank A.F., Fernandes R.P.M., Scher R. (2012). Leishmanicidal activity of carvacrol-rich essential oil from *Lippia. sidoides* Cham. Biol. Res..

[32] Osorio E., Arango G., Robledo S., Muñoz D., Jaramillo L., Vélez I. (2006). Antileishmanial and cytotoxic activity of synthetic aromatic monoterpens. Acta Farm. Bonaer..

[33] Medeiros M.G.F., Silva A.C., Citó A.M.G.L., Borges A.R., Lima S.G., Lopes J.A.D., José A.D.L., Regina C.B.Q.F. (2011). In vitro antileishmanial activity and cytotoxicity of essential oil from *Lippia sidoides* Cham. Parasitol. Int..

[34] Ueda-Nakamura T., Mendonça-Filho R.R., Morgado-Díaz J.A., Maza P.K., Dias Filho B.P., Cortez D.A.G., Alviano D.S., Nakamura C.V. (2006). Antileishmanial activity of Eugenol-rich essential oil from *Ocimum. gratissimum*. Parasitol. Int..

[35] Dutra F.L., Oliveira M.M., Santos R.S., Silva W.S., Alviano D.S., Vieira D.P., Lopes A.H. (2016). Effects of linalool and eugenol on the survival of *Leishmania. (L.) infantum chagasi* within macrophages. Acta Trop..

[36] Dorman H.J.D., Deans S.G. (2000). Antimicrobial agents from plants: antibacterial activity of plant volatile oils. J. Appl. Microbiol..

[37] Nazzaro F., Fratianni F., De Martino L., Coppola R., De Feo V. (2013). Effect of essential oils on pathogenic bacteria. Pharmaceuticals.

[38] Machado M., Dinis A.M., Santos-Rosa M., Alves V., Salgueiro L., Cavaleiro C., Sousa M.C. (2014). Activity of Thymus capitellatus volatile extract, 1,8-cineole and borneol against *Leishmania.* species. Vet. Parasitol..

[39] Rosa M.S.S., Mendonça-Filho R.R., Bizzo H.R., Rodrigues I.A., Soares R.M.A., Souto-Padrón T., Alviano C.S., Lopes A.H. (2003). Antileishmanial Activity of a Linalool-Rich Essential Oil from *Croton cajucara*. Antimicrob. Agents Chemother..

[40] Zheljazkov V.D., Cantrell C.L., Tekwani B., Khan S.I. (2008). Content, composition, and bioactivity of the essential oils of three basil genotypes function of harvesting. J. Agric. Food Chem..

[41] Camargos H.S., Moreira R.A., Mendanha S.A., Fernandes K.S., Dorta M.L., Alonso A. (2014). Terpenes increase the lipid dynamics in the Leishmania plasma membrane at concentrations similar to their IC_50_ values. PLoS ONE.

[42] Rios M.C., Silva W.R.T., Azevedo A.F., Santos P.L., Teixeira S.A., Muscará M.N., Thomazzi S.M., Almeida R.P., Fernandes R.P.M., Scher R. (2015). Expression of glyceraldehyde 3-phosphate dehydrogenase is enhanced in *Leishmania.* spp. naturally resistant to nitric oxide. Genet. Mol. Res..

[43] Azevedo A.F.A., Dutra J.L.L., Santos M.L.B., Santos D.A., Alves P.B., Moura T.R., Almeida R.P., Fernandes M.F., Scher R., Fernandes R.P.M. (2014). Fatty acid profiles in *Leishmania.* spp. isolates with natural resistance to nitric oxide and trivalent antimony. Parasitol. Res..

[44] Giudice A., Camada I., Leopoldo P.T.G., Pereira J.M.B., Riley L.W., Wilson M.E., John L.H., de Jesus A.R., Edgar M.C., Roque P.A. (2007). Resistance of *Leishmania. (Leishmania.) amazonensis* and *Leishmania. (Viannia.) braziliensis* to nitric oxide correlates with disease severity in Tegumentary Leishmaniasis. BMC Infect. Dis..

[45] Rodrigues F.A.R., Bomfim I.S., Cavalcanti B.C., Pessoa C., Goncalves R.S.B., Wardel J.L., Wardell S.M., de Souza M.V. (2014). Mefloquine–Oxazolidine Derivatives: A New Class of Anticancer Agents. Chem. Biol. Drug Des..

[46] Winter R. (2009). A Consumer's Dictionary of Food Additives-Descriptions in Plain English of More than 12,000 Ingredients both Harmful and Desirable Found in Foods.

[47] Robledo S., Osorio E., Muñoz D., Jaramillo L.M., Restrepo A., Arango G., Vélez I. (2005). In Vitro and In Vivo Cytotoxicities and Antileishmanial Activities of Thymol and Hemisynthetic Derivatives. Antimicrob. Agents Chemother..

[48] Arruda D.C., Miguel D.C., Yokoyama-Yasunaka J.K., Katzin A.M., Uliana S.R. (2009). Inhibitory activity of limonene against Leishmania parasites in vitro and in vivo. Biomed. Pharmacother..

[49] Kulshrestha A., Bhandari V., Mukhopadhyay R., Ramesh V., Sundar S., Maes L. (2013). Validation of a simple resazurin-based promastigote assay for the routine monitoring of miltefosine susceptibility in clinical isolates of *Leishmania. donovani*. Parasitol. Res..

[50] International Standard ISO 10993–5 (2009). MTT Cytotoxicity Test. Biological Evaluation of Medical Devices-Part 5: Tests for In Vitro Cytotoxicity.

